# The Latest Studies on Lotus (*Nelumbo nucifera*)-an Emerging Horticultural Model Plant

**DOI:** 10.3390/ijms20153680

**Published:** 2019-07-27

**Authors:** Zhongyuan Lin, Cheng Zhang, Dingding Cao, Rebecca Njeri Damaris, Pingfang Yang

**Affiliations:** 1 State Key Laboratory of Biocatalysis and Enzyme Engineering, School of Life Sciences, Hubei University, Wuhan 430062, China; 2 Key Laboratory of Plant Germplasm Enhancement and Specialty Agriculture, Wuhan Botanical Garden, Chinese Academy of Sciences, Wuhan 430074, China; 3 University of Chinese Academy of Sciences, Beijing 100039, China

**Keywords:** *Nelumbo nucifera*, phylogeny, genomics, molecular mechanisms, model plant

## Abstract

Lotus (*Nelumbo nucifera*) is a perennial aquatic basal eudicot belonging to a small family *Nelumbonaceace*, which contains only one genus with two species. It is an important horticultural plant, with its uses ranging from ornamental, nutritional to medicinal values, and has been widely used, especially in Southeast Asia. Recently, the lotus obtained a lot of attention from the scientific community. An increasing number of research papers focusing on it have been published, which have shed light on the mysteries of this species. Here, we comprehensively reviewed the latest advancement of studies on the lotus, including phylogeny, genomics and the molecular mechanisms underlying its unique properties, its economic important traits, and so on. Meanwhile, current limitations in the research of the lotus were addressed, and the potential prospective were proposed as well. We believe that the lotus will be an important model plant in horticulture with the generation of germplasm suitable for laboratory operation and the establishment of a regeneration and transformation system.

## 1. Introduction

**Figure 1:**
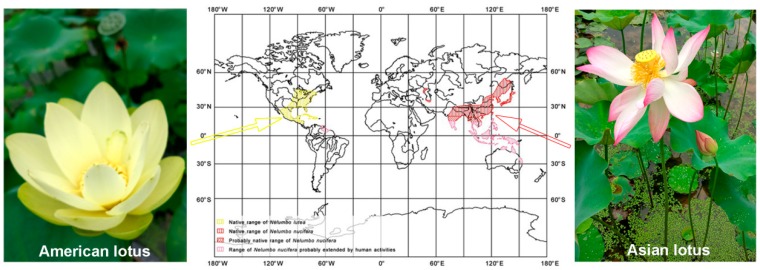
Overview of the lotus species and their global distribution. The left and right panels show the flowers of American and Asian lotus, respectively. The yellow and red shadow areas in the world map of the middle panel show the distributions of American and Asian lotus, respectively. (Figure revised from Li, Y. et al. [4]).

Lotus is a perennial aquatic plant. It belongs to the small family of *Nelumbonaceae*, comprising of only one genus Nelumbo with two species: *Nelumbo nucifera* Gaertn. and *Nelumbo lutea* Pear., which are popularly named as Asian lotus and American lotus, respectively [1]. Generally, lotus refers to Asian lotus and mainly distributes in Asia and the north of Oceania, while the American lotus primarily occurs in the eastern and southern parts of North America, as well as the north of South America [1,2,3,4] (Figure 1). Being separated by the Pacific Ocean, these two species differ in their external morphologies, such as petal color and shape, leaf shape and plant size [5] (Figure 1). In spite of this, both of them have the same chromosome number (2n = 16), and show a similar life style, with about five months of life span for each generation. Crossing between these two species could generate an F1 population, which is totally infertile. Although there are only two species of lotus in taxonomy, very abundant germplasms exist all over the world, which display variable genetic backgrounds and phenotypes, especially in Asia. In addition, the lotus is a basal eudicot, which makes it a very important species in plant phylogenetic and evolution studies.

Asian lotus is also named as sacred lotus because of its significance in the religions of Buddhism and Hinduism [5]. It is a very good symbol in Chinese traditional culture. All of these make sacred lotus a very popular ornamental plant. In addition, it is also a popular vegetable and traditional medicinal plant with great economic value in South-East Asia. China is regarded as one of the major centers in lotus cultivation and breeding, with over several thousands of years of cultivation history [1]. As the result of the long period of breeding, domestication and cultivation, large amounts of lotus cultivars have been obtained, showing variable morphology and other traits. The cultivated lotus is generally divided into three categories, namely, rhizome, seed and flower lotus, according to their usage in reality. The lotus rhizome and seed could not only be consumed as vegetables, but are also used for lotus propagation, whereas, the flower lotus is mainly applied in ornamentation and environmental improvement. Based on the climatic regions they are accustomed to, sacred lotus could also be classified into two ecotypes, which are temperate lotus and tropical lotus. The temperate lotus has an enlarged rhizome occurring after flowering and its leaves wither. In contrast, the tropical lotus has a whip-like rhizome with a longer green period and flowering time [1,2]. 

Because of its importance in horticulture, medicinal usage and plant phylogeny, the sacred lotus has gained increasing interests from the scientific community. It will undoubtedly enhance the breeding and application of lotus to obtain enough fundamental knowledge about this plant. Recently, the genome of two sacred lotus germplasms were sequenced and released [6,7], which facilitates further study on this species. Up-to-date, there are nearly 1000 research publications focusing on different aspects of the lotus, half of which were published in the last decade. In this review, we summarized the latest advancement of studies on the sacred lotus in order to provide a comprehensive insight into the basic biology and economic usage of this important plant, which might also contribute to future studies on lotus breeding and germplasm enhancement.

## 2. Phylogeny and Genomic Studies

Taxonomically, lotus belongs to the genus of *Nelumbo*, which is the only existing genus of the Nelumbonaceae family. Cretaceous fossils have been assigned to Nelumbonaceae. Analysis on these fossils indicate that the family of Nelumbonaceae might have more than 100 Ma years of history, and showed considerable morphological stasis. [8]. Determination of lotus classification in taxonomy took a long time. Because of its superficial similarities in the flowers and vegetative body with the waterlily, Nelumbo used to be regarded as one genus of the Nymphaeaceae family in the old classification system. In the Cronquist system, the Nelumbonaceae family was recognized, but still placed in the order of Nymphaeales [9]. In both the Dahlgren system [10] and the Thorne system [11], the Nelumbonaceae family was placed in its own order, Nelumbonales. Takhtajan [12] removed Nelumbonaceae from Nymphaeales, but placed them alone in the subclass of the Nelumbonidae. With the increasing accumulation of evidence at the molecular level, The Angiosperm Phylogeny Group (APG) has placed it into the basal eudicot order of Proteales, which is outside of the core eudicots (http://www.mobot.org/MOBOT/research/APweb/, last accessed date: 23 June, 2019) [13].

Except for Nelumbonaceae, Proteales contains three other families, including Platanaceae, Proteaceae, and Sabiaceae, of which the former two are the closest relatives of the lotus, and are mainly shrubs and woody trees [14], indicating the possibility of the lotus being a land plant adapted to aquatic environments. Interestingly, the family of Nelumbonaceae is still classified within the order of Nymphaeales on the USDA webpage (https://plants.sc.egov.usda.gov/core/profile?symbol=NENU2, last accessed date: 22 July, 2019). Furthermore, studies also showed that the gene expression patterns in the floral organs of *Nymphaea* and *Nelumbo* are remarkably similar to each other [15]. It would be interesting to understand the evolutionary convergences between Nymphaeales and the lotus.

From the genetic point of view, both species of lotus are diploid with the number of chromosomes 2n = 16. The predicted size of the lotus genome is 929 Mb, which is based on the flow cytometry analysis [16]. In 2013, the draft genomes of two lotus wild germplasms ‘China Antique’ and ‘Chinese Tai-zi’ were sequenced, assembled and released [6,7], which made lotus into a model angiosperm along with the other 22 species (http://www.mobot.org/MOBOT/research/APweb/trees/modeltreemap.html, last accessed date: 23 June, 2019). The assembled genome size of ‘China Antique’ is 804 Mb and the sequencing data shows that this genome contains 26,685 protein-coding genes [6]. Recently, a more comprehensive transcriptomic analysis increased the number of protein-coding genes to 32,121 in ‘China Antique’ [17]. The assembled genome of ‘Chinese Tai-zi’ is 792 Mb with 36,385 protein-coding genes [7,18]. Based on their data, it seems that the lotus genome contains a high content of repeat sequences, with transposable elements (TEs) accounting for about 50% of the genome sequence. The availability of these two genomes will facilitate further studies on the different biological features of lotus, including agronomic and horticultural traits, and might contribute to the knowledge of flowering plant evolution. Wang et al. [19] combined the lotus genome and transcriptome data of ‘China Antique’ and constructed the public accessible lotus genome database (http://lotus-db.wbgcas.cn, last accessed date: 20 March, 2015), which makes further molecular and genetic studies on this species more convenient among the scientific community. Additionally, the lotus chloroplast and mitochondrion genome were also sequenced, which have been applied in optimizing the genetic maps and analyzing the evolution of the lotus [20,21]. Because of the availability of abundant genome information, phylogenetic and evolution analysis of lotus at the molecular level was also conducted, which showed the functional divergence of miRNAs in temperate and tropical lotus [22]. Based on the study, 57 pre-eudicot miRNA families from different evolutionary stages were predicted. Combining the miRNA data and the lotus genome information, it was revealed that the loss of miRNA families in descendent plants is associated with that in duplicated genomes [22]. However, because of the high percentage of repetitive sequences (>47%), the assembly of the lotus genome, especially for ‘China Antique’, is still far behind completion, although a study has been conducted aiming at anchoring the megascaffolds into eight chromosomes [23]. The nine anchored megascaffolds, which have a combined size of 543.4 Mb, just account for 67.6% of the lotus genome. The advent of a third generation sequencing technique has been successfully applied in many other species, which will be able to improve the assembly of this lotus genome in the near future.

## 3. Unique Properties of Lotus

Biologically, lotus has not only the common aquatic plant features, but also certain unique features that distinguish it from other plant species. These features include seed longevity, leaf ultrahydrophobicity and floral thermoregulation. Understanding of the mechanisms that lead to the formation of these unique properties is important, for not only the basic plant biology, but also their great usage potential through bionics. 

Lotus fruit is famous for its longevity. It was reported that lotus fruits buried underground over 1300 years in the Northeast of China could still be germinated [24]. Understanding the underlying mechanism of lotus seed longevity may contribute to enhancing seed storage in agriculture, and even in the healthcare of human beings. 

Previous studies have shown that the first factor contributing to this feature might be the chemical compositions of lotus fruit wall, which contains high contents of polysaccharides (galactose, mannose) and tannins [25]. These compounds might help to prevent any negative effects from the environment. Recently, another study showed that the polyphenols content in lotus seed epicarp increased along with the ripening, and showed strong anti-oxidation activity [26], which might also be helpful. Besides of the physical factors, several thermo-proteins, which showed high stability under high temperature, were also indicated to be helpful. These proteins include CuZn-SOD, 1-CysPRX, dehydrin, Cpn20, Cpn60, HSP80, EF-1α, Enolase1, vicilin, Met-Synthase and PIMT [27]. The functions of some genes involved in seed thermos-tolerance and germination vigor, including *NnANN1* and *NnPER1* (*Peroxiredoxin 1*), were verified in transgenic *Arabidopsis* [28,29]. To achieve this, the lotus genome contains multi-copies for most of the antioxidative genes [6,7]. Recent study showed that small RNA might also be involved in the regulation of lotus seed longevity [30]. How these different factors cooperatively function to contribute to the lotus seed longevity is still elusive, but worthy of studying. More importantly, it is very interesting to know if these factors also work in other systems.

Lotus leaves exhibit ultra-hydrophobicity, which is also known as the “lotus effect” [31]. This characteristic of ultra-hydrophobicity could ensure that the leaf upper epidermis is not covered by water, thus maintaining the normal function of its stomata [32]. Because of this, ultrahydrophobicity is believed to be an advantage in the evolution of the lotus. Studies have shown that it is achieved by a special dense layer of waxy papillae on the lotus leaf surface [33,34]. Further studies showed that the easily rolling water droplets could help to remove the dirt particles adhering on the leaf surface and result in a self-cleaning phenomenon, which is heavily dependent upon the contact angle [35]. Two wax biosynthesis-related genes (*NnCER2* and *NnCER2*-*LIKE*) were cloned from the lotus, and transformed in *Arabidopsis*, which resulted in an alteration of the cuticle wax structure in inflorescence stems, and proved their function in the biosynthesis of the extra-long fatty acids [36]. More studies on the lotus leaf chemical compositions and structure might be very helpful in producing materials with super-hydrophobicity and self-cleaning features.

In addition, floral organ thermogenesis is another unique feature of the lotus, which independently occurs at receptacle, stamen and petal, respectively [37]. This property has been proven to be the results of a cyanide-resistant alternative oxidase pathway conducted in the floral organs [38,39,40], which initiated extensive studies on alternative oxidases (AOXs) and plant uncoupling mitochondrial proteins (PUMPs) [41]. This feature of thermogenesis seems to be ecologically important for the sexual reproduction of the lotus through attracting insect pollinators [42]. Studies have shown that the generated heat could either provide a warm environment to the thermo-sensitive pollinators or help to release the volatile compounds to attract the flying insects, mainly beetles [37,43,44]. Generation of heat only occurs before anthesis, which ends with pollination and a fertilized ovary. After anthesis, there is no need to attract the pollinators any more, and the main function of the floral organs, especially the receptacle, transits into photosynthesis [45,46]. It will be very important to explore the mechanism that controls this kind of metabolism transition.

## 4. Genetic and Molecular Studies on the Horticultural Traits of the Lotus

**Figure 2:**
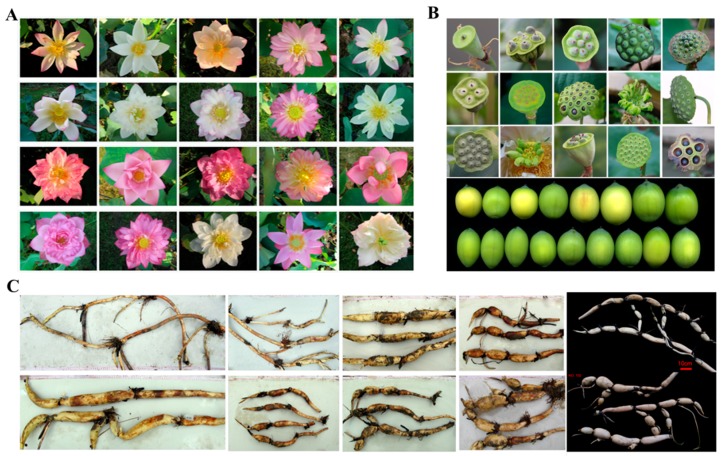
The diversified phenotypes of the Asian lotus germplasm. (**A**) Flower lotus germplasm showing different flower color and shape. (**B**) Seed lotus germplasm showing different size and shape of seed and seedpod. (**C**) Rhizome lotus germplasm showing different branching, elongation and expansion of the rhizome.

As mentioned above, a lotus is also a popular ornamental, vegetable and medicinal plant, with great potential of utilization in reality, based on which, three types of lotus, named as flower, seed, and rhizome lotus, were defined. Each type of lotus shows notable abundant variable phenotypes (Figure 2), which provide suitable germplasm for its breeding and further study on different traits. Recently, a number of studies have been conducted focusing on the genetic and molecular mechanisms underlying the formation of different traits of lotus flower, seed and rhizome. These traits could largely determine the economic value of the lotus, hence becoming the main factors selected in its breeding. Several genetic maps have been constructed through crossing between different germplasms with contrasting phenotypes in some of the economic traits, based on which a number of molecular markers associated with the target traits were developed, including ISSR, AFLP, SSR, RAPD, and SRAP [47,48,49,50]. 

Meanwhile, whole genome re-sequencing on the natural germplasm also identified abundant SNPs and Indels [51,52,53]. Together, these data will undoubtedly facilitate the lotus breeding.

### 4.1. The Flower of Lotus

**Figure 3:**
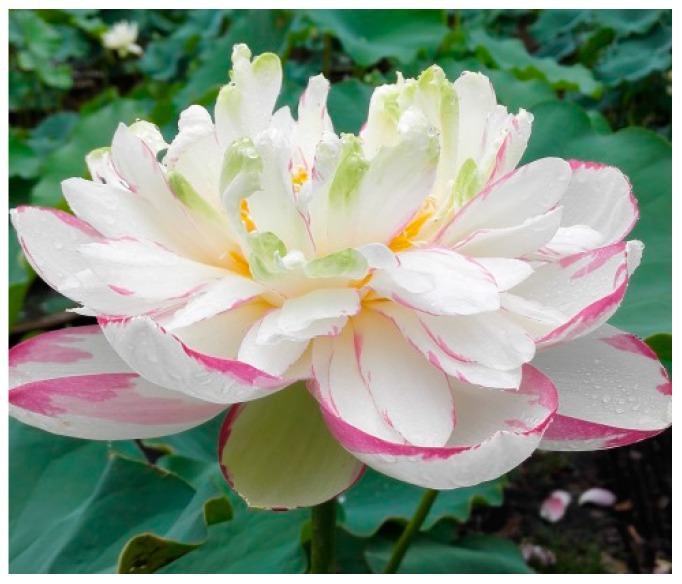
Lotus cultivar with spotted color flower.

Lotus flower is among the top ten traditional famous flowers in China, and was chosen as the national flower in India and Vietnam. It is widely cultivated for its aesthetic value, which is largely attributed to its gorgeous color and its diversified form and shape (Figure 2). For ornamental plants, flower color and shape are the major two factors that determine their ornamental value. The lotus petals show three major colors; white, red and yellow, with the former two existing only in Asian lotus and the later one only in American lotus. Through breeding and artificial selection, many cultivars with mixed colors have been obtained on the purpose of increasing its ornamental value (Figure 2). A large-scale analysis on the pigment composition of different germplasm has shown that the yellow and red color is mainly determined by the contents of carotenoids and anthocyanins, respectively [54]. Genome-wide analysis of the *MYB* gene family indicated that there is a similar anthocyanin biosynthesis regulatory system in lotus and *Arabidopsis* [55], based on which an overexpression of *NnMYB5* in *Arabidopsis* resulted in the accumulation of anthocyanin in immature seeds and flower stalks [56]. In spite of this similarity, a comparative proteomics study between white and red cultivars showed that the expression of the *ANS* gene might be the major reason for the absence of anthocyanin biosynthesis in the white flower lotus [57]. Further analysis found that different levels of methylation occur in the promoter regions of *ANS* gene between the two cultivars, which indicates the epigenetic regulation on expression of this gene. However, the gene that lead to the different methylation level on the promoter of *ANS* gene between the red and white lotus cultivars is still unknown. In addition, there are cultivars showing genetic constant spotted color (Figure 3), which is still not understood. It will be very important not only to the breeding of flower lotus, but also to enriching our knowledge on the coloration of plant flowers to explore the mechanism underlying the regulation of spotted color in lotus.

In addition to color, flower shape is also important for the economic value of ornamental plants. Based on different purposes of breeding, lotus cultivars with diversified flower shapes were obtained, including few-petalled, semidouble-petalled, double-petalled, duplicate-petalled and all-double-petalled cultivars [2]. For the semidouble-petalled, double-petalled shapes, they are usually the resultants of stamen petaloid. Comparative transcriptomic studies among petal, stamen petaloid and stamen through RNA-seq were conducted, which identified several candidate genes involved in stamen petaloid, especially some MADS-box genes [58]. Their study revealed 11 MADS-box genes and one *APETALA2* (*AP2*) gene being involved in the stamen petaloid phenomenon. Among them, *AGL15*, *AGL80* and *AGAMOUS* genes are positively related to, and *AGL6* is negatively related to the stamen petaloid [58]. Meanwhile, a genome-wide DNA methylation analysis was also conducted among these three tissues, which indicates the potential involvement of epigenetic regulation on the stamen petaloid [59]. However, this study did not detect any obvious changes of the methylation on the MADS-box genes [59]. There also exist pistil petaloid cultivars (Figure 2), in which the stamen petaloid also occurs. How these are coordinately regulated is still unknown in the lotus. Furthermore, it is well known that lotus bloom in the summer days, which brings some challenges for its wide utilization in ornamentation. It will be very important to make it bloom either earlier or later for ornamental purposes. Hence, unveiling the mechanism controlling the time of flowering is also important. A transcriptomic analysis has been conducted aiming at exploring the candidate genes that control the time of flowering, which indicate the existence of a complicated regulatory network [60]. Their data indicate that the differential regulation of some photoperiod related genes, such as *COP1, CCA1, LHY, CO-LIKE*, and *FT*, the vernalization gene *VIN3* and the gibberellic acid-related gene *GAI*, might be involved in the regulation of early flowering in lotus. Specifically, several isoforms of the *FT* gene were found to be differentially expressed [60]. 

### 4.2. Rhizome and Seeds

As mentioned above, lotus is not only an ornamental plant, but also a vegetable because of its edible rhizome and seeds. Lotus has a morphologically modified subterraneous stem. Especially for the temperate ecotype, its subterraneous stem is enlarged in autumn, which is known as rhizome (Figure 2). The rhizome contains abundant starch, proteins and vitamins, making it a popular edible vegetable. Enlargement of lotus rhizome could largely determine its economic value. In addition, the enlarged rhizome could also help the lotus to survive from winter during its bud dormancy, and provide substrates and energy for its asexual propagation. This phenomenon is very similar with the tuberization of the potato, which has been proven to be regulated through a very intricate genetic network. Being a significant feature distinguishing between the temperate and tropical lotus, it may also facilitate in understanding the evolution and domestication of the lotus [2,61]. It seems that rhizome enlargement is tightly related to the flowering in a lotus. Usually, the enlargement occurs after flowering. For the purposes of increasing its yield in agricultural production, genetic and transcriptomic studies focusing on the enlargement of this rhizome have been conducted. 

Gene expressions during the rhizome development were analyzed through RNA-Seq, which identified the specific candidate genes for rhizome enlargement [62]. The results also indicated the role of SNPs and alternative splicing (AS) events in Asian lotus rhizome development [61,63]. Similar with the yield traits in many crops, the enlargement of the lotus rhizome is a quantitative trait. Developing a suitable genetic population and constructing high density genetic map will be very helpful to elucidate the mechanism underlying rhizome development and enlargement.

Besides its longevity, lotus seed is also edible either fresh or dry matured, with an additional medicinal versatility resulting from compounds like alkaloids, flavonoids and certain micronutrients [3,5]. Both the size and number of the seeds per seedpod vary among different lotus cultivars (Figure 2). It is very important to increase its nutrition as well as its yield in lotus seed production. To achieve this, comparative proteomics and metabolomics studies were conducted on lotus seeds during its development, which not only deepen the understanding on the development of lotus seed, but also determine candidate genes crucial for lotus seed size [62]. In addition, comparative transcriptomic analysis was also conducted between two lotus germplasms with contrasting phenotypes in both seed size and seed number per seedpod [64]. Similar to rhizome, the yield of seed is also a quantitative trait, which requires more study at the genetic aspect. Meanwhile, because of its medicinal usage, it is necessary to conduct a comprehensive analysis on it metabolites during seed development.

### 4.3. Secondary Metabolites and Medicinal Usage of Lotus

Lotus is a traditional herb, of which nearly each tissue has a medicinal usage [65,66,67]. It has been used as a traditional Chinese medicine for over a thousand years. This might ascribe to its abundant content of secondary metabolites, including flavonoids, phenolic acids and alkaloids [65,66,67]. Systematic studies were conducted in optimizing the method to extract these metabolites from different tissues of the lotus [26,54,68,69,70,71,72,73,74,75,76,77]. Meanwhile, distributions of different secondary metabolites in different tissues of lotus were profiled [26,54,68,69,70,71,72,73,74,75,76,77]. Furthermore, assessment of the lotus germplasm with different origins was also performed by these established methods [65,66,67], which helped in screening of the germplasm with a high content of specific secondary metabolites. These candidate germplasms might be used for either the breeding or for further study on the biosynthesis of different metabolites in the lotus. In addition, the potential medicinal usage of different lotus secondary metabolites was also assessed [65,66,67]. However, the exact compounds that function in each medicinal usage are still unknown, which seems to be the general challenge for most traditional Chinese medicine.

Specifically, the leaf of a lotus is a very important traditional Chinese herbal medicine, which has been widely used in controlling the blood lipids and treating hyperlipidemia [78]. In the last decade, it is becoming more and more popular as weight-losing tea in China to reduce the level of lipids in the human body [79]. Studies have shown that alkaloids are the major bioactive compounds in lotus leaves, with nuciferine and N-nornuciferine being the major two [80,81,82]. To evaluate the biosynthesis pathway of alkaloids and its regulation in lotus leaf, several transcriptomic studies were performed [83,84], which revealed that a benzylisoquinoline alkaloids (BIA) biosynthetic pathway and its transcriptional regulation differ in high BIAs lotus compared with low BIAs lotus [84]. Several genes encoding the enzymes involved in the BIA biosynthetic pathway were proposed based on sequence similarity analysis [85]. Further functional analysis of these genes will be necessary to obtain comprehensive knowledge on the biosynthesis of these bioactive compounds.

### 4.4. Studies on the Establishment of Lotus Regeneration and Transformation System

To be a model horticultural plant, it might be necessary to establish a transformation system, which will facilitate the studies on the functions of different genes in the lotus. A study was conducted to induce the formation of a callus from different explants of the lotus, in which somatic embryo cultivated in suitable medium containing a combination of different growth regulators was proposed [86]. To obtain more in-depth understanding, a proteomic analysis was conducted to identify the key proteins that might be critical for the induction of callus from developing cotyledon [87]. 

Directly inducing the formation of a shoot from the bud has also been successfully performed [88]. Based on this system, various studies have been conducted to transform the lotus. It seems that the induced shoot from the embryo apical bud could be successfully transformed through a particle bombardment device with a pCAMBIA2301 vector [89]. This method not only succeeded with the GUS reporter gene, but also with the anti-sense of two anthocyanin biosynthesis genes *dihydroflavonol 4-reductase* (anti-*DFR*) and *Chalcone synthase* (anti-*CHS*) [89,90]. Except for the group from Thailand, there are still no other studies conducted successfully on the transformation of the lotus, although a lot of researchers are working on this. It seems there are still challenges on the reproducibility and the efficiency of the transformation, as well as the selection of a suitable cultivar.

## 5. Conclusions and Perspectives

Because of its significance in the ordinary life of the population in South and East Asia, as well as in horticultural and medicinal usage, lotus is attracting more and more attention from the scientific community. A large number of studies have been conducted on nearly all aspects of this plant, including phylogeny and evolution, genomics, genetics and breeding and medicinal usage. With the release of its genome information, -omics and molecular genetics studies, focusing on the economic traits of this plant have stepped into the center, which undoubtedly will contribute a lot to the lotus breeding. Unfortunately, there are still some limitations that constrain the studies, especially the molecular biology study, on this species. The first one might be the assembly and annotation of its genome, which still needs to be improved further. Secondly, there is no universally recognized lotus cultivar or germplasm that is commonly used for the basic biology studies in the scientific community. Among all the germplasm, the sequenced one ‘China Antique’ might be an ideal candidate because of its genetic homozygosity. Thirdly, the low efficiency of the regeneration and transformation system seriously prevents the molecular genetic studies on the lotus, which is a prerequisite for gene function study. The fourth, but not the last, is the indeterminate growth and long life span (~5 months per generation) of the lotus plant, which limits the cultivation of lotus in small space. Through artificial selection, a number of cultivars with small plant architecture and short life span (~3 months’ generation time) were obtained in lotus, which are very popular in the ornamental market, and named as ‘Wan Lian’ (bowl lotus). To cross these bowl lotus with ‘China Antique’, and then subject to backcrossing breeding, it might be possible to obtain germplasm with both small plant size and the ‘China Antique’ genetic background. This type of germplasm might be suitable for cultivation in the lab, and hence for further studies at molecular level. In conclusion, lotus could be regarded as an emerging model of horticultural plants, and be capable for the utilization in studying many aspects of unique features in plants.

## Abbreviations

AFLP	Amplified Fragment Length Polymorphism
AGL	AGAMOUS-like
ANN	Annexin
ANS	anthocyanin synthase
AOX	alternative oxidases
APG	The Angiosperm Phylogeny Group
AS	alternative splicing
BIA	benzylisoquinoline alkaloid
CCA	CIRCADIAN CLOCK ASSOCIATED
CER	ECERIFERUM
CHS	Chalcone synthase
CO-LIKE	CONSTANS-like
COP	CONSTITUTIVELY PHOTOMORPHOGENIC
Cpn	Chaperonin
DFR	dihydroflavonol 4-reductase
EF	elongation factor
HSP	Heat shock protein
FT	FLOWERING LOCUS T
GAI	gibberellic acid insensitive
ISSR	inter-simple sequence repeat
LHY	LATE ELONGATED HYPOCOTYL
MADS-box	MINICHROMOSOME MAINTENANCE 1 (MCM1), AGAMOUS (AG), DEFICIENS (DEF), and SERUM RESPONSE FACTOR (SRF) domain
PIMT	Protein L-isoaspartyl methyltransferase
PRX	Peroxiredoxin
PUMP	plant uncoupling mitochondrial protein
RAPD	Random Amplified Polymorphic DNA
SNP	single nucleotide polymorphism
SOD	superoxide demutase
SRAP	Sequence—related amplified polymorphism
SSR	Simple Sequence Repeats
TE	transposable element
VIN3	vernalization
